# New structural insights into podocyte biology

**DOI:** 10.1007/s00441-017-2590-3

**Published:** 2017-03-10

**Authors:** Florian Grahammer

**Affiliations:** 1 Department of Medicine IV, Medical Center and Faculty of Medicine, University of Freiburg, Breisacherstrasse 66, 79106 Freiburg, Germany; 2 III. Medical Clinic, University Medical Center Hamburg-Eppendorf, Martinistrasse 52, 20246 Hamburg, Germany

**Keywords:** Glomerulum, Podocyte, Glomerular basement membrane, Slit diaphragm, Renal filtration

## Abstract

The last 5 years have witnessed tremendous advances in both light- and electron-microscopic techniques in the biomedical sciences. Application of these new cutting-edge methods to glomerular biology has advanced considerably and, in part, completed our endeavor to draw a detailed map of the glomerular tuft. The scope of this review is to illustrate these new insights within both the morphometry of podocyte cells and the architecture of the glomerular filtration barrier and to assess whether these findings have indeed had an impact on our biological understanding of glomerular function.

## Introduction

For centuries, the elucidation of the challenging three-dimensional (3D) architecture of the kidney has paved the way for a very fruitful interaction between making morphologic discoveries on the one hand and probing their functional consequences on the other (Berliner 1995; Furukawa et al. 1991; Navar 2004). A major driver of this process has been the constant development of better microscopic techniques that seemingly culminated with the invention of the electron microscope in the 1930s, allowing the inspection of structures below the resolution of light-based systems (approximately 200 nm). Transmission (TEM) and later on scanning (SEM) electron microscopy enabled the examination of the fine structures of the glomerulum and, especially, the visualization of the fragile cell-cell contacts between neighboring podocyte foot processes, the so-called slit-diaphragm (SD; Fujita et al. 1970; Rinehart et al. 1953; Yamada 1955). Although stimuli from ultrastructural discoveries became scarce between 1980 and 2005, the last decade has spurred innovative new imaging techniques that are now increasingly used in the field of glomerular biology and hence that boost the biological translation of new morphologic discoveries.

**Fig. 1:**
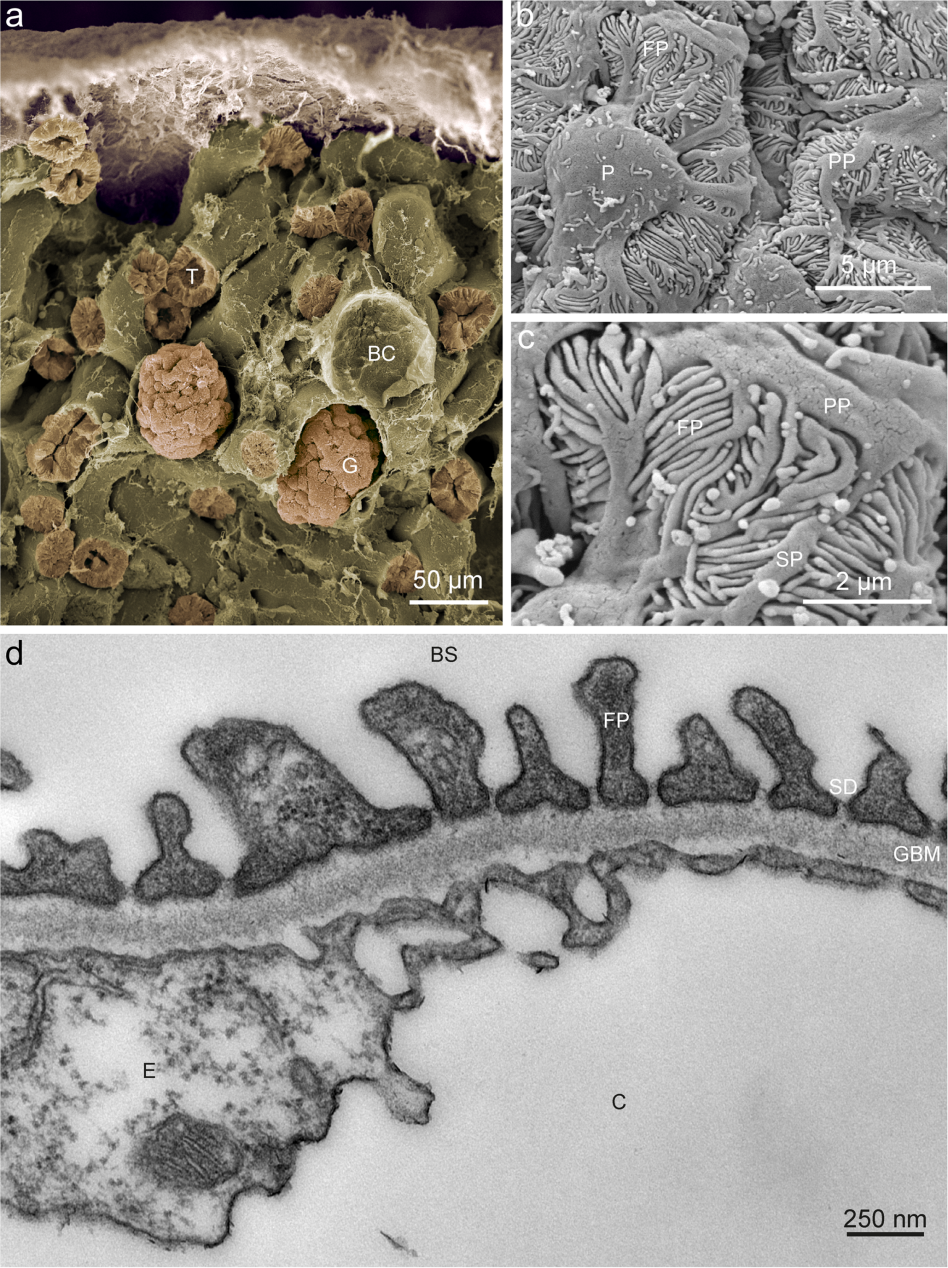
Glomerular ultrastructure. **a** False-colored low-resolution scanning electron micrograph of mouse renal cortex displaying several tubuli (*T*) and two glomeruli (*G*). In addition, an empty Bowman’s capsule (*BC*) can be seen from which the glomerulum was lost during preparation. **b** Detailed scanning electron micrograph showing a podocyte (*P*), primary processes (*PP*) originating from the cell body and foot processes (*FP*). **c** High-resolution scanning electron micrograph illustrating primary processes (*PP*), secondary processes (*SP*) and foot processes (*FP*). **d** Transmission electron micrscopy of a mouse glomerular capillary (*C*) revealing the structural composition of the glomerulus. The endothelial cell (*E*) coats the inner surface of the capillary wall and is followed by the three layers of the glomerular basement membrane (*GBM*). On the outside of the capillary, the foot processes (*FP*) cover a major part of the GBM circumference. The slit-diaphram can be discerned in between the foot processs. The primary filtrate drains into Bowman’s space (*BS*) and, via the urinary pole, reaches the proximal tubule

Podocytes reside as specialized pericytes covering the glomerular capillary endothelial cells towards the parietal epithelial cells that coat Bowman’s capsule (Fig. 1a; Greka and Mundel 2012). Together with the endothelial cells of the capillary, podocytes build-up the glomerular basement membrane (GBM), which seems to be the decisive filtration layer within the glomerular filtration barrier (GFB; Caulfield and Farquhar 1974). In addition to their cell body, podocytes consist in long primary processes that interdigitate with each other forming the so-called foot processes (or secondary processes), as became evident with the first SEM studies of mouse and human glomerula (Fig. 1b, c; Arakawa 1971; Fujita et al 1970). Together, primary and foot processes cover the major part of the capillary surface. Within neighboring foot processes, a peculiar and specialized cell-cell junction forms, namely the SD (Fig. 1d; Grahammer et al. 2013b). Several podocyte hallmark proteins, e.g., NEPHRIN, NEPH1 and PODOCIN, take part in SD formation and generate the broadest known mammalian cell-cell contact (Grahammer et al. 2013a).

Although we have a working concept of glomerular filtration today, several potentially decisive details fully explaining this paramount process have not been discerned to a desirable degree. Whereas molecular approaches have identified a multitude of novel, seemingly important genes and proteins, these newly identified molecules have, so far, only modestly advanced our overall understanding of GFB, because of the complexity of the involved processes (Boerries et al. 2013). With the advent of new imaging techniques, we now have a good chance of elucidating these mechanisms further.

## Structural advances

### Development of podocytes

Glomerular development is a highly regulated process and has been reviewed in detail elsewhere (Quaggin and Kreidberg 2008; Schell et al. 2014). Several conserved signaling cascades drive the interaction between the branching of the ureteric bud, the condensation of the metanephric mesenchym, the formation of the renal vesicle and the subsequent sequences leading to a glomerulus and a tubular apparatus. Podocyte precursors form a simple columnar epithelium with individual cells being connected by tight and adherence junctional complexes. During further development, this apically located junctional complex moves towards the basolateral aspects of the podocytes. With increasing formation of the foot processes and the interdigitation of the podocytes, the molecular composition of the cell-cell junctions changes towards that of the final SD, which finally replaces the immature junctions. Although histology, immunofluorescence techniques and TEM have considerably helped us to understand the principles of renal development, an ultrastructural 3D analysis of the sequential developmental changes to podocytes has so far been lacking. Recently, the EM techniques of serial block-face SEM (SBF-SEM) and focused ion beam SEM (FIB-SEM) have become available, both of which allow the 3D reconstruction of ultrastructural images with unprecedented speed and accuracy (Denk and Horstmann 2004; Heymann et al. 2006). Excitingly, the application of the FIB-SEM technique has led to the discovery of so far unreported details during glomerular development (Ichimura et al. 2017). Whereas, during the S-shape body stage, podocyte precursors have the known cobblestone appearance from the luminal side, their basolateral surface is irregularly shaped with the first cytoplasmic protrusions being inserted under neighboring podocytes. These protrusions appear to be independent of any tight, adherence, or SD junctional complexes. As expected, the junctional complex itself moves downwards from the upper 25% of the cell to its basal aspects. Interestingly, tricellular portions of these adherence and tight junctional complexes form the leading edge. Whether this propagation of junctional movement is a function linked to the specialized proteins of tricellular junctions (e.g., ILDR1, ILDR2 and LSR), some of which are expressed in isolated podocytes, needs further investigations (Boerries et al. 2013; Higashi et al. 2013). In the capillary loop stage, primitive primary and foot processes are formed, the latter still being devoid of any junctional processes. Finally, at the maturing glomerulum stage, junctional complexes, initially of adherence and tight junctional composition, can be seen between the short and irregular foot processes but are gradually replaced by SD-like junctions. Meanwhile, ridge-like prominences develop at the base of primary foot processes, which find their way under neighboring podocytes, thereby separating their cell bodies from the GBM and creating the subpodocyte space. Once matured, primary processes form a cytoplasmic arcade that is often situated in the valleys between adjacent capillary loops (Ichimura et al. 2017).

### Mature glomeruli

A comparison of the results of FIB-SEM with those of classic TEM in mature glomerula in adult rat kidney revealed that the former, because of reduced osmium tetroxide staining, are especially useful for visualizing membrane-containing organelles, e.g., the endoplasmic reticulum (ER) or Golgi apparatus, whereas the podocyte actin cytoskeleton is much better depicted by the latter (Ichimura et al. 2015). FIB-SEM allows the segmentation and 3D reconstruction of large glomerular volumes. In contrast to previous reports, primary processes do not seem to be connected to the GBM by the full sole of their basal aspect but rather are only attached to the GBM by a ridge-like prominence (Burghardt et al. 2015; Ichimura et al. 2015). Foot processes from neighboring podocytes connect to this structure by their distal ends. In addition, this 3D ultrastructural analysis shows that foot processes not only emerge from primary processes but can also originate directly from the cell body (Burghardt et al. 2015; Ichimura et al. 2015). Intriguingly, in this case, a ridge-like prominence seems to be the nascent point for foot processes. The FIB-SEM technique has helped to answer the decade-long question as to whether foot processes of the same podocyte interact with each other (Burghardt et al. 2015). Only foot processes of neighboring podocytes seem to form interdigitating structures, not foot processes belonging to the same cell.

A detailed comparative analysis of the GFB ultrastructure by using TEM, SBF-SEM, FIB-SEM and TEM tomography was recently performed (Arkill et al. 2014). A new lanthanum-based dye stained the glycocalyx of the endothelium and podocytes with superior resolution compared with tannic-acid-based protocols. The width of the endothelial glycocalyx seems to be one order of magnitude broader than that of the podocyte plasma membrane. In addition, a densly stained luminal glycocalyx compartment is present that contrasts with a sparsely stained compartment close to the endothelial cell wall. Whether this finding has some biological relevance or is just a matter of dye penetrance needs further clarification. Whereas several distinct subpodocyte spaces with differing characteristics have been demonstrated, their physiological relevance in concert with the position of endothelial fenestrae and podocyte slits needs considerable additional research efforts (Arkill et al. 2014).

In a similar way to EM techniques, sub-diffraction light microscopy has experienced tremendous advancements during the last decade (Arkill et al. 2014; Hell 2007). Nanoscopy of the GBM in health and disease has provided so far unprecedented molecular details of the structural assembly of the broadest basement membrane within our body. Whereas conventional immunofluorescence images always show one layer of AGRIN, stochastic optical reconstruction microscopy (STORM) has resolved two distinct layers of this protein approximately 130 nm apart (Suleiman et al. 2013). This has been confirmed by using AGRIN c-term antibodies directed against various epitopes. Antibodies against AGRIN n-term have also shown two layers but with a reduced distance of 108 nm. This might be an indication that AGRIN molecules are positioned in an oblique fashion within the GBM. The centrally oriented n-terminal binds the coiled-coil domain of LAMININ1, whereas the peripherally oriented c-terminal points in the direction of the respective adjacent cell layers. Applying the same technique, other important ECM proteins within or adjacent to the GBM, e.g., INTEGRIN β1, LAMININ 521, COLLAGEN IV and NIDOGEN, were mapped (Suleiman et al. 2013).

Although all the above has been determined in murine tissue, the authors also performed some of their mapping approach to human specimens. Based on previous data, all previously mentioned ECM molecules should also be present within the human GFB, although the human GBM is approximately twice as thick as its murine counterpart (Hudson et al. 2003). Interestingly, this increased thickness is mainly attributable to enlarged COLLAGEN IV networks and two additional layers of LAMININ 521, which were positioned towards the central aspect of the GBM. Based on their distance to the neighboring cell layers, these two layers seem unlikely to interact directly with INTEGRIN molecules (Suleiman et al. 2013).

Not only has the fine structure and composition of the GBM attracted considerable attention over the last years but also, to a similar degree, the molecular alignment, function and make-up of the SD (Grahammer et al. 2013b). From the traditional view based on Rodewald’s and Karnovsky’s seminal work in 1974 and the cloning of Nephrin in 1998 by the Tryggvason group, NEPHRIN molecules from either side of the foot process are thought to overlap to form a zipper-like structure with an electron-dense midline (Rodewald and Karnovsky 1974; Ruotsalainen et al. 1999). In between the individual molecules, holes smaller than the size of ALBUMIN form and restrict passage of this marker molecule (Tryggvason et al. 2006; Wartiovaara et al. 2004). Despite intriguing findings from freeze-substitution EM studies in the 1980s challenging the zipper theory, this became the widely accepted concept of the SD (Furukawa et al. 1991; Ohno et al. 1992; Tryggvason et al. 2006).

Two recent publications now question the traditional SD view, nicely confirming the results obtained by freeze substitution 30 years earlier (Burghardt et al. 2015; Grahammer et al. 2016). Using different species, fixation and EM techniques, both groups can show that the SD complex consists in more than one layer of molecules. Examination of fixed tissue specimens with EM tomography has led to the identification of one conventional SD-like layer of molecules with punctate cell-cell contacts on top and filamentous cell-cell contacts below (Burghardt et al. 2015). Application of EM tomography to high-pressure frozen or freeze-substituted murine specimens has demonstrated a multilayered bipartite SD scaffold. Within this scaffold, all molecules span the width of the filtration slit and only very occasionally form an electron-dense midline (in approximately 2.5% of cases). Shorter molecules with a median extracellular domain (ECD) length of 19 nm are situated in several layers (∼3-5) towards the GBM, whereas longer molecules (ECD approximately 43 nm) form loose layers (∼2) towards Bowman’s space (Grahammer et al. 2016). The width spanned by the bottom layer corresponds well to the ECD of NEPH1, whereas that of the top layer fits nicely with the ECD of NEPHRIN. Based on the amorphous structure of the SD, we consider it unlikely that this presents a considerable filtration barrier to molecules that have passed the GBM. Although its precise function, despite these exciting findings, remains unknown, the homology of its building blocks (NEPH1 and NEPHRIN) to TITIN and other molecules functioning as molecular springs indicate that, in contrast to current thinking, the task of the SD is rather to keep the foot processs apart than to tie them together. On a pathomechanistic level, the apical movement of the SD, as is observed frequently under pathological conditions, can be interpreted as the removal of the spring or spacer that consequently leads to foot process effacement, the characteristic podocyte damage response. Subsequent studies with atomic force microscopy or related techniques will have to establish whether both NEPH1 and NEPHRIN indeed act as molecular springs keeping foot processes apart.

### Pathomechanisms

Light microscopy, immunofluorescence and standard TEM are routine techniques used in a thorough assessment of biopsy samples of patients with proteinuric kidney diseases. Application of advanced imaging techniques to mainly murine disease models might reveal disease-specific features that so far have escaped pathological evaluation. An examination of *Collagen IV* Alport mice with STORM microscopy revealed a split of the two layers of AGRIN molecules in parts of the GBM. In addition, the distribution of COLLAGEN α1α1α2 is no longer restricted to the endothelial lamina rara of the GBM but can be found dispersed throughout the GBM of these animals (Suleiman et al. 2013). Using the same Alport disease model at different timepoints, another group elucidated the pathognomonic features of the podocyte GBM interaction by applying SBF-SEM (Randles et al. 2016). Compared with age-matched control animals, Alport mice exhibit increasing areas of reduced foot process density. In addition, cellular invasion originating from podocytes and invading the GBM can be discerned. Interestingly, these protrusions were not limited to the model of Alport disease in which the remodeling of the GBM is a central pathomechanistic process but also occurred in *Myo1e* and *Ptpro* knock-out mice, models for respectively human SRNS/FSGS disease (Randles et al. 2016). This indicates that the invasion of the GBM by foot processes is of broader pathologic relevance and might be an attempt to improve the fixation of podocytes at the GBM and to prevent podocyte loss.

At a molecular level, the process of podocyte foot process effacement is still embarrassingly poorly understood. Although the process of effacement and its potential reversibility has been described by means of TEM in both investigational and clinical papers since the middle of the 1970s, no generally accepted pathophysiologic concept for foot process effacement exists to date (Caulfield et al. 1976; Robson et al. 1974; Seiler et al. 1975). Whereas intercellular tight junctions can be frequently observed by TEM in disease states, integral components of tight junctions can be detected at veritable amounts under physiologic conditions (Fukasawa et al. 2009). Whether these proteins form part of the native unchallenged SD complex, or whether they form an emergency stock of tight junction proteins ready to replace the SD in the potential case of foot process effacement remains unclear (Fukasawa et al. 2009). Interestingly, in the rat puromycin aminonucleoside nephrosis model, the expression of pre-existing tight junction proteins is increased being supplemented by the de novo expression of further members of this protein family (Fukasawa et al. 2009; Gong et al. 2017). The transgenic overexpression of CLAUDIN1 in adult mice leads to the destabilization of the SD complex, the formation of tight junctions between foot processs and the onset of proteinuria at 2 weeks after the start of doxycycline treatment (Gong et al. 2017). Advanced protein localization studies are warranted if we are to improve our understanding and to potentially influence the molecular sequences leading to foot process effacement.

## Concluding remarks

Advanced imaging techniques have yielded exciting new structural insights into glomerular architecture over the last couple of years. Although we are still trying to grasp the biological relevance and consequences of these findings, new technical adventures are appearing on the horizon. For example, the extension of FIB-SEM to pre-embedding immunogold studies might allow us to reconstruct molecular trafficking pathways within a cell. Concepts of glomerular filtration could potentially be established by combining endogenous fluorescently labeled marker proteins in transgenic mice with advanced correlative light- and electron-microscopic techniques (Johnson et al. 2015). Helium ion SEM technology has the power to provide superb new insights into the topographical ultrastructure of the glomerulum (Rice et al. 2013). Lastly, an improvement of the available clearance techniques might enhance our possibilities to localize proteins in health and disease when combined with high- or super-resolution microscopy (Richardson and Lichtman 2015; Unnersjo-Jess et al. 2016).

## References

[CR1] Arakawa M (1971). A scanning electron microscope study of the human glomerulus. Am J Pathol.

[CR2] Arkill KP, Qvortrup K, Starborg T, Mantell JM, Knupp C, Michel CC, Harper SJ, Salmon AH, Squire JM, Bates DO, Neal CR (2014). Resolution of the three dimensional structure of components of the glomerular filtration barrier. BMC Nephrol.

[CR3] Berliner RW (1995). Homer Smith: his contribution to physiology. J Am Soc Nephrol.

[CR4] Boerries M, Grahammer F, Eiselein S, Buck M, Meyer C, Goedel M, Bechtel W, Zschiedrich S, Pfeifer D, Laloe D, Arrondel C, Goncalves S, Kruger M, Harvey SJ, Busch H, Dengjel J, Huber TB (2013). Molecular fingerprinting of the podocyte reveals novel gene and protein regulatory networks. Kidney Int.

[CR5] Burghardt T, Hochapfel F, Salecker B, Meese C, Gröne HJ, Rachel R, Wanner G, Krahn MP, Witzgall R (2015). Advanced electron microscopic techniques provide a deeper insight into the peculiar features of podocytes. Am J Physiol Renal Physiol.

[CR6] Caulfield JP, Farquhar MG (1974). The permeability of glomerular capillaries to graded dextrans. Identification of the basement membrane as the primary filtration barrier. J Cell Biol.

[CR7] Caulfield JP, Reid JJ, Farquhar MG (1976). Alterations of the glomerular epithelium in acute aminonucleoside nephrosis. Evidence for formation of occluding junctions and epithelial cell detachment. Lab Invest.

[CR8] Denk W, Horstmann H (2004). Serial block-face scanning electron microscopy to reconstruct three-dimensional tissue nanostructure. PLoS Biol.

[CR9] Fujita T, Tokunaga J, Miyoshi M (1970). Scanning electron microscopy of the podocytes of renal glomerulus. Arch Histol Jpn.

[CR10] Fukasawa H, Bornheimer S, Kudlicka K, Farquhar MG (2009). Slit diaphragms contain tight junction proteins. J Am Soc Nephrol.

[CR11] Furukawa T, Ohno S, Oguchi H, Hora K, Tokunaga S, Furuta S (1991). Morphometric study of glomerular slit diaphragms fixed by rapid-freezing and freeze-substitution. Kidney Int.

[CR12] Gong Y, Sunq A, Roth RA, Hou J (2017). Inducible expression of claudin-1 in glomerular podocytes generates aberrant tight junctions and proteinuria through slit diaphragm destabilization. J Am Soc Nephrol.

[CR13] Grahammer F, Schell C, Huber TB (2013). Molecular understanding of the slit diaphragm. Pediatr Nephrol.

[CR14] Grahammer F, Schell C, Huber TB (2013). The podocyte slit diaphragm—from a thin grey line to a complex signalling hub. Nat Rev Nephrol.

[CR15] Grahammer F, Wigge C, Schell C, Kretz O, Patrakka J, Schneider S, Klose M, Arnold SJ, Habermann A, Brauniger R, Rinschen MM, Volker L, Bregenzer A, Rubbenstroth D, Boerries M, Kerjaschki D, Miner JH, Walz G, Benzing T, Fornoni A, Frangakis AS, Huber TB (2016). A flexible, multilayered protein scaffold maintains the slit in between glomerular podocytes. JCI Insight.

[CR16] Greka A, Mundel P (2012). Cell biology and pathology of podocytes. Annu Rev Physiol.

[CR17] Hell SW (2007). Far-field optical nanoscopy. Science.

[CR18] Heymann JA, Hayles M, Gestmann I, Giannuzzi LA, Lich B, Subramaniam S (2006). Site-specific 3D imaging of cells and tissues with a dual beam microscope. J Struct Biol.

[CR19] Higashi T, Tokuda S, Kitajiri S, Masuda S, Nakamura H, Oda Y, Furuse M (2013). Analysis of the “angulin” proteins LSR, ILDR1 and ILDR2—tricellulin recruitment, epithelial barrier function and implication in deafness pathogenesis. J Cell Sci.

[CR20] Hudson BG, Tryggvason K, Sundaramoorthy M, Neilson EG (2003). Alport’s syndrome, Goodpasture’s syndrome, and type IV collagen. N Engl J Med.

[CR21] Ichimura K, Miyazaki N, Sadayama S, Murata K, Koike M, Nakamura K, Ohta K, Sakai T (2015). Three-dimensional architecture of podocytes revealed by block-face scanning electron microscopy. Sci Rep.

[CR22] Ichimura K, Kakuta S, Kawasaki Y, Miyaki T, Nonami T, Miyazaki N, Nakao T, Enomoto S, Arai S, Koike M, Murata K, Sakai T (2017). Morphological process of podocyte development revealed by block-face scanning electron microscopy. J Cell Sci.

[CR23] Johnson E, Seiradake E, Jones EY, Davis I, Grunewald K, Kaufmann R (2015). Correlative in-resin super-resolution and electron microscopy using standard fluorescent proteins. Sci Rep.

[CR24] Navar LG (2004). The legacy of Homer W. Smith: mechanistic insights into renal physiology. J Clin Invest.

[CR25] Ohno S, Hora K, Furukawa T, Oguchi H (1992). Ultrastructural study of the glomerular slit diaphragm in fresh unfixed kidneys by a quick-freezing method. Virchows Arch B.

[CR26] Quaggin SE, Kreidberg JA (2008). Development of the renal glomerulus: good neighbors and good fences. Development.

[CR27] Randles MJ, Collinson S, Starborg T, Mironov A, Krendel M, Konigshausen E, Sellin L, Roberts IS, Kadler KE, Miner JH, Lennon R (2016). Three-dimensional electron microscopy reveals the evolution of glomerular barrier injury. Sci Rep.

[CR28] Rice WL, Van Hoek AN, Paunescu TG, Huynh C, Goetze B, Singh B, Scipioni L, Stern LA, Brown D (2013). High resolution helium ion scanning microscopy of the rat kidney. PLoS One.

[CR29] Richardson DS, Lichtman JW (2015). Clarifying tissue clearing. Cell.

[CR30] Rinehart JF, Farquhar MG, Jung HC, Abul-Haj S (1953). The normal glomerulus and its basic reactions in disease. Am J Pathol.

[CR31] Robson AM, Giangiacomo J, Kienstra RA, Naqvi ST, Ingelfinger JR (1974). Normal glomerular permeability and its modification by minimal change nephrotic syndrmone. J Clin Invest.

[CR32] Rodewald R, Karnovsky MJ (1974). Porous substructure of the glomerular slit diaphragm in the rat and mouse. J Cell Biol.

[CR33] Ruotsalainen V, Ljungberg P, Wartiovaara J, Lenkkeri U, Kestila M, Jalanko H, Holmberg C, Tryggvason K (1999). Nephrin is specifically located at the slit diaphragm of glomerular podocytes. Proc Natl Acad Sci U S A.

[CR34] Schell C, Wanner N, Huber TB (2014). Glomerular development—shaping the multi-cellular filtration unit. Semin Cell Dev Biol.

[CR35] Seiler MW, Venkatachalam MA, Cotran RS (1975). Glomerular epithelium: structural alterations induced by polycations. Science.

[CR36] Suleiman H, Zhang L, Roth R, Heuser JE, Miner JH, Shaw AS, Dani A (2013). Nanoscale protein architecture of the kidney glomerular basement membrane. elife.

[CR37] Tryggvason K, Patrakka J, Wartiovaara J (2006). Hereditary proteinuria syndromes and mechanisms of proteinuria. N Engl J Med.

[CR38] Unnersjo-Jess D, Scott L, Blom H, Brismar H (2016). Super-resolution stimulated emission depletion imaging of slit diaphragm proteins in optically cleared kidney tissue. Kidney Int.

[CR39] Wartiovaara J, Ofverstedt LG, Khoshnoodi J, Zhang J, Makela E, Sandin S, Ruotsalainen V, Cheng RH, Jalanko H, Skoglund U, Tryggvason K (2004). Nephrin strands contribute to a porous slit diaphragm scaffold as revealed by electron tomography. J Clin Invest.

[CR40] Yamada E (1955). The fine structure of the renal glomerulus of the mouse. J Biophys Biochem Cytol.

